# PD-L1, PD-1, LAG-3, and TIM-3 in Melanoma: Expression in Brain Metastases Compared to Corresponding Extracranial Tumors

**DOI:** 10.7759/cureus.6352

**Published:** 2019-12-11

**Authors:** Jaeyun Jane Wang, Peter Burger, Janis Taube, Abha Soni, Kaisorn Chaichana, Mary Sheu, Zineb Belcaid, Christopher Jackson, Michael Lim

**Affiliations:** 1 Neurosurgery, Johns Hopkins University School of Medicine, Baltimore, USA; 2 Pathology, Johns Hopkins University School of Medicine, Baltimore, USA; 3 Dermatology, Johns Hopkins University School of Medicine, Baltimore, USA

**Keywords:** melanoma, immune checkpoint markers, tumor microenvironment, metastasis, programmed death-ligand 1 (pd-l1), programmed cell death receptor 1 (pd-1), lymphocyte activation gene 3 (lag-3), t-cell immunoglobulin and mucin-domain containing-3 (tim-3)

## Abstract

Background

Metastatic melanoma to the brain carries a particularly poor prognosis that may be associated with an attenuated antitumor response in the presence of central nervous system malignancies. Thus, the development of brain metastases could theoretically accelerate cancer progression both locally and systemically. Although dysregulation of checkpoint markers, such as programmed death-ligand 1 (PD-L1), programmed cell death receptor 1 (PD-1), lymphocyte activation gene 3 (LAG-3), and T-cell immunoglobulin and mucin-domain containing-3 (TIM-3), have been implicated in immune dysfunction, the exact relationship between these markers and brain tumor-mediated immune suppression remains unclear. Thus, the objective of this study was to explore whether there exists a differential expression of the above checkpoint markers in the intracranial milieu as compared to tumors in the periphery, which may shed light on the mechanism behind the diminished antitumor response.

Methods

We identified nine patients with extracranial melanomas and matched intracranial metastases. Formalin-fixed, paraffin-embedded slides were stained for PD-L1, PD-1, LAG-3, and TIM-3 via immunohistochemistry. Qualitative analysis was performed to assess the staining of the markers in the neoplastic and lymphocytic cells, which were the two cell lineages in each biopsy.

Results

Expression of PD-1 and TIM-3 between extracranial and intracranial tumoral sites was conserved. Specifically, in lymphocytes, PD-1 expression was observed in 100% of extracranial and 100% of intracranial slides, whereas TIM-3 expression was seen in 33.33% of extracranial and 33.33% of intracranial slides. Neither marker stained tumor cells, as expected. PD-L1 showed a slight variation in staining between sites, with lymphocyte staining in 100% of extracranial and 88.89% of intracranial slides, and the same percentages per site for tumor cells. The greatest variability was observed in LAG-3 lymphocyte staining, with staining in 77.78% of extracranial and 33.33% of intracranial slides. No LAG-3 staining of tumor cells was noted, as expected.

Conclusion

Preliminary analysis revealed the conservation of PD-L1, PD-1, LAG-3, and TIM-3 expression intra- and extracranially. This could suggest that these markers are important in maintaining an immunosuppressive phenotype at both sites. Another possibility is that this pattern of expression is associated with patients who develop brain metastasis, as this was the only subset of patients included in this study. Interestingly, LAG-3 staining of lymphocytes appeared more prominent in extracranial over intracranial tumors. Future studies should include more samples to draw out potential patterns masked by the small sample size, as well as to compare checkpoint expression in other patient groups, such as those with non-brain metastasis or those with no metastasis at all.

## Introduction

Melanoma is a neoplasm of melanocytes that is associated with significant morbidity and mortality with the 2018 American Cancer Society estimates for the United States pointing to 91,270 new diagnoses and 9,320 deaths [1]. Notably, the most common cause of melanoma-related mortality is from brain metastases, with the overall median survival dropping to well under one year with the development of brain involvement [2-6].

Of course, central nervous system (CNS) metastases are known to be a relatively late event in disease progression; thus, the survival outcomes could be attributed to a manifestation of late-stage disease rather than brain-specific involvement. However, interestingly, melanoma patients who experience brain metastases fare worse than those who have non-brain visceral metastases. Specifically, the probability of death in patients with brain metastases is twice, seven times, and 12 times higher than in patients with gastrointestinal, lung, and lymph node or subcutaneous metastasis, respectively [2]. This suggests that CNS involvement is not merely a marker of disease progression but rather independently impacts survival.

While the exact mechanism behind this phenomenon is unknown, a diminished immune response against CNS tumor antigens is likely involved. Specifically, not only is the CNS often designated as immunologically unique, as it is characterized by the existence of the blood-brain barrier and a relative scarcity of antigen-presenting cells [7-8], but a recent study by Jackson et al. showed that melanoma brain metastases may diminish the immune response against tumor antigens and thus hasten the progression of systemic disease [9]. An example of this immunosuppression in CNS malignancies is the development of T-cell exhaustion, which is characterized by such dysfunction as the inability to proliferate or failure to secrete cytokines in response to antigen stimulation.

More broadly, this phenomenon can be observed in both cancer and chronic infection, and various checkpoint molecules have been associated with such immunosuppression. Specifically, programmed death-ligand 1 (PD-L1), programmed cell death receptor 1 (PD-1), lymphocyte activation gene 3 (LAG-3), and T-cell immunoglobulin and mucin-domain containing-3 (TIM-3) have all been implicated in cluster of differentiation (CD)-8+ T-cell exhaustion [10-17], although the precise role of these markers in brain-tumor-mediated immunosuppression is unclear. Thus, it would be interesting to assess whether the T-cell dysfunction associated with CNS involvement is reflected by differential expression of various checkpoint markers in the intracranial milieu as compared to tumors in the periphery.

In brief, melanoma metastasis to the brain carries a poor prognosis that goes beyond marking late-stage disease, and this may in part be explained by immune suppression in the context of CNS involvement. The exact mechanism behind this brain-tumor-mediated immune suppression is unknown, although such phenomena as T-cell exhaustion have been implicated. In addition, although various checkpoint markers have been associated with T-cell dysfunction, the exact relationship between CNS-associated immune dysfunction and these markers remains unclear. Thus, our current study explores whether the expression of four checkpoint markers (PD-L1, PD-1, LAG-3, and TIM-3) differs in extracranial melanomas versus the subsequent brain metastases, which may shed further light on the mechanism behind CNS-mediated immune suppression.

## Materials and methods

Patient selection

The study protocol was approved by the Johns Hopkins Institutional Review Board. Forty-six patients with pathologically diagnosed melanoma who underwent resection of brain metastases between 2008 and 2017 at Johns Hopkins Hospital were identified. In this particular study, intracranial tumors were defined as metastatic melanoma to the brain, while extracranial tumors were defined as either a primary melanoma tumor or non-brain metastatic melanoma. We required that biopsies of both the extracranial and intracranial melanoma be available on site and found nine patients who matched this criterion.

Study design

Hematoxylin and eosin (H&E) slides of each tumor biopsy block were used to identify the optimal section of a tumor to stain. Formalin-fixed, paraffin-embedded slides were created from the chosen blocks and evaluated for PD-L1, PD-1, LAG-3, and TIM-3 expression. Qualitative analysis (positive versus negative staining) was performed to assess the staining of the markers in tumor and lymphocyte cells, which were the two cell lineages present in each biopsy.

Immunohistochemistry staining

The slides of interest were deparaffinized and rehydrated in a series of alcohols. Antigen retrieval was performed in a decloaking chamber (Biocare Medical, Pacheco, CA) at 120° C with citrate buffer at pH 6.0 (Dako S1699, Agilent Technologies, Inc., Santa Clara, CA) for 10 minutes. This was followed by peroxidase, protein, avidin, and biotin blocking. The primary antibody was incubated overnight at the following concentrations for each antigen of interest: 1) PD-L1 (SP142, Spring Bioscience, Abcam, Cambridge, MA) at 1:800 dilution for a final concentration of 0.1 ug/mL and rabbit IgG control (MA516385, Life Technologies, Carlsbad, CA) at a final concentration of 0.1 ug/mL; 2) PD-1 (NAT105, Abcam, Cambridge, MA) at 1:1,000 dilution for a final concentration of 1 ug/mL; 3) LAG-3 (17B4, LifeSpan Biosciences, Seattle, WA) at a final concentration of 0.1 ug/mL; 4) TIM-3 (F38-2E2, Affymetrix, Inc., Santa Clara, CA) at a final concentration of 2 ug/mL.

## Results

Patient demographics

Of the initial 46 patients identified, those who did not have biopsies of both their primary tumor and brain metastases at JHH were excluded, as they did not have tissue readily available for staining. Thus, a total of nine patients were included in this study. Demographics are summarized in Table 1. 

**Table 1:** Patient Demographics

Patient	Gender	Age at initial biopsy	Location of extracranial tumor	Location of brain metastasis	Time between biopsies (days)	Immunotherapy (prior to brain biopsy)
1	Male	47	Left flank	Left frontal lobe	936	IL-1, nivolumab, IL-21
2	Male	80	Left posterior scalp	Left frontal lobe	251	None
3	Female	57	Right thigh	Right parietal lobe	132	Interferon a-2b
4	Female	50	Lung, right middle lobe	Right frontal lobe	85	Vemurafenib
5	Male	70	Right axillary lymph node	Right occipital lobe	413	None
6	Male	29	Intraparotid lymph node	Left frontal lobe	77	None
7	Female	51	Colonic lymph node	Left occipital lobe	194	None
8	Male	73	Lung, right upper lobe	Right parietal lobe	143	None
9	Male	82	Left temporal scalp	Right frontal lobe	81	None

Overall, of the nine patients, six were male and three were female. The average age at the initial biopsy of the extracranial tumor was 59.89 years old (standard deviation (SD): 17.58), and the average time between biopsies of the extracranial and intracranial tumors was 256.89 days (SD: 276.35).

Immunohistochemical staining

**Figure 1:**
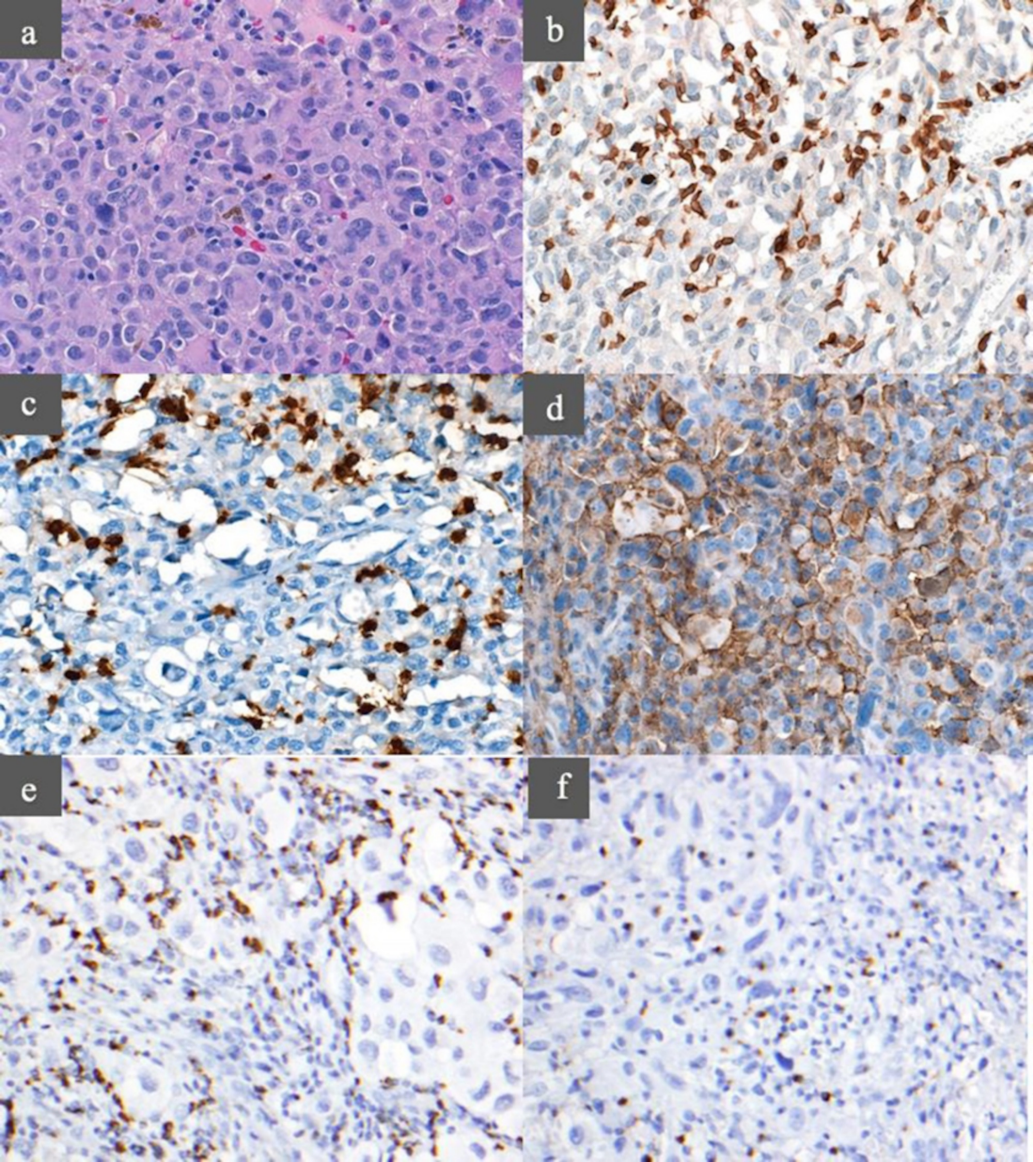
Immunohistochemical staining a) Hematoxylin and eosin (H&E); b) cluster of differentiation 3 (CD-3); c) programmed cell death protein 1 (PD-1); d) programmed death-ligand 1 (PD-L1); e) lymphocyte-activation gene 3 (LAG-3); f) T-cell immunoglobulin and mucin-domain containing-3 (TIM-3)

H&E stains of all slides were prepared to provide a baseline image of the tissue (Figure 1a). Images showed small, round cells infiltrating the tumor. CD-3 staining verified that T-cells composed the infiltrative cell population in the tumor (Figure 1b). The remaining images show representative staining patterns of each of the four checkpoint markers, with the brown color representing the presence of antigen (Figure 1c-1f).

Staining analysis

**Figure 2:**
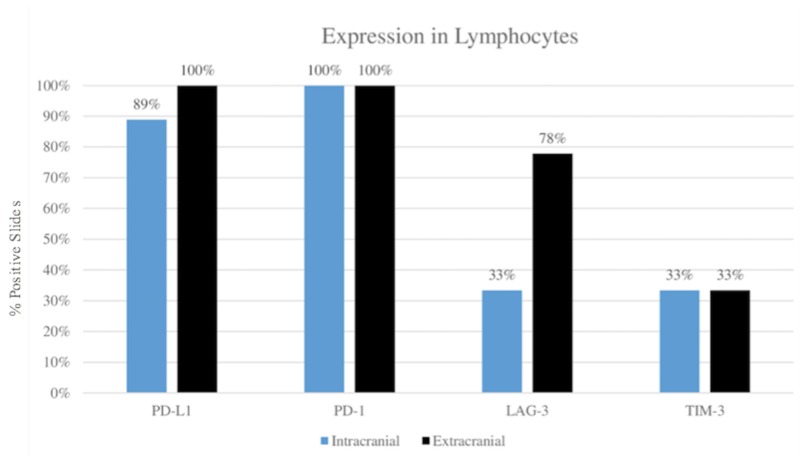
Comparison of immune checkpoint marker expression between extracranial and intracranial tumor sites in lymphocytes LAG-3: lymphocyte activation gene 3; PD-1: programmed cell death receptor 1; PD-L1: programmed death-ligand 1; TIM-3: T-cell immunoglobulin and mucin-domain containing-3

**Figure 3:**
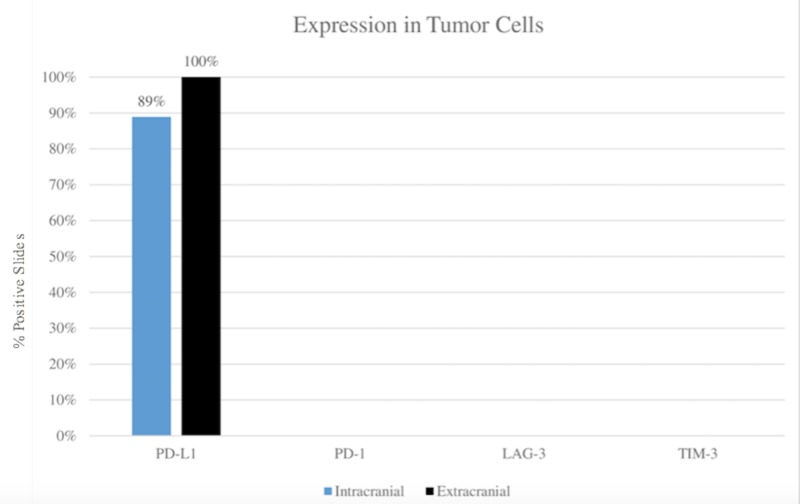
Comparison of immune checkpoint marker expression between extracranial and intracranial tumor sites in tumor cells LAG-3: lymphocyte activation gene 3; PD-1: programmed cell death receptor 1; PD-L1: programmed death-ligand 1; TIM-3: T-cell immunoglobulin and mucin-domain containing-3

As shown in Figures 2-3, visualization and scoring of the stained slides on light microscopy revealed no variation in expression of PD-1 and TIM-3 between tumor sites. Specifically, PD-1 stained lymphocytes in 100% of extracranial and 100% of intracranial slides, while TIM-3 stained lymphocytes in 33.33% of extracranial and 33.33% of intracranial slides. Neither marker stained tumor cells, as expected.

PD-L1 showed a slight variation in staining between sites, with lymphocyte staining in 100% of extracranial and 88.89% of intracranial slides, and the same percentages per site for tumor cells. The greatest variability was observed in LAG-3 lymphocyte staining, with staining in 77.78% of extracranial and 33.33% of intracranial slides. No LAG-3 staining of tumor cells was noted, as expected.

## Discussion

Malignant melanoma is known to metastasize to the brain and carries a poor prognosis when it reaches the CNS. A retrospective review conducted by Seigler et. al revealed that the overall median survival of 702 patients who were identified with clinically significant brain metastases was a mere 113.2 days [4]. Although brain metastasis itself is indeed a marker of late-stage disease, it also leads to worse overall survival compared to metastases to other organs, perhaps indicating that there is something unique about CNS involvement.

This poor prognosis can be attributed in part to the host’s attenuated antitumor response in the presence of a CNS malignancy, which could theoretically accelerate cancer progression both locally and systemically. Separately, dysregulation of checkpoint markers, such as PD-L1, PD-1, LAG-3, and TIM-3, have been implicated in immune suppression, although the exact relationship of these markers to brain tumor-mediated immune suppression remains unclear. Thus, to further elucidate why melanoma brain metastases carry such a poor prognosis, we were interested in detecting potential differences in checkpoint expression between extracranial and intracranial melanoma.

Ultimately, analysis of the stained slides showed conservation of PD-L1, PD-1, LAG-3, and TIM-3 expression between intracranial and extracranial samples. Interestingly, LAG-3 staining of lymphocytes appeared more prominent in extracranial tumors.

Several possible explanations can be given regarding these findings. Firstly, the general homogeneity in expression between tumor sites could suggest that these four markers are important in maintaining the immunosuppressive phenotype at both sites. Thus, perhaps there is another marker not included in this study that can be associated with the immunosuppression seen with CNS involvement. Secondly, there may be a significant discrepancy in LAG-3 expression between tumor locations that could not be detected by this study due to the small sample size.

Finally, this study focused only on melanoma patients with CNS metastasis, which means that this pattern of checkpoint expression could be associated with this particular subset of patients. For example, TIM-3 expression was identical between tumor sites but was overall quite low relative to markers, such as PD-L1 and PD-1. Thus, perhaps low TIM-3 expression in the context of high PD-L1 and PD-1 expression is associated with the development of CNS metastasis specifically but is not seen in the development of non-CNS metastasis.

A few limitations should be considered when interpreting the results of this study. The small sample size of nine patients limits our ability to detect trends as well as to stratify the group by patient and disease characteristics. Specifically, we were also unable to divide the patients based on any concurrent immunotherapy they received and analyze whether that had an impact on expression patterns. In addition, analysis of the staining was qualitative, as each slide was assigned “positive” or “negative” for staining. Thus, we were unable to analyze the results based on staining intensity. Of note, because we used horseradish peroxidase for the immunohistochemistry, we would not be able to quantify staining intensity using this particular study protocol.

However, this study ultimately provided a preliminary view into patterns of immune checkpoint marker expression based on tumor location. Future studies should incorporate a wider range of markers beyond the four included in this report, as well as increase the study population, so as to better power the analysis. Comparing expression patterns across more specific subgroups of patients, such as those with a primary tumor only, those who developed non-CNS metastasis, and those who developed CNS metastasis, could also potentially reveal further trends.

## Conclusions

In this study, we explored whether there exists a differential expression of PD-L1, PD-1, LAG-3, and TIM-3 in the intracranial environment as compared to tumors in the periphery. Tissue samples from nine patients were stained for these checkpoint markers via immunohistochemistry. Preliminary analysis revealed conservation of PD-L1, PD-1, LAG-3, and TIM-3 expression intra- and extracranially, although LAG-3 staining of lymphocytes appeared more prominent in extracranial tumors.
